# Apelin Does Not Impair Coronary Artery Relaxation Mediated by Nitric Oxide-Induced Activation of BK_Ca_ Channels

**DOI:** 10.3389/fphar.2021.679005

**Published:** 2021-05-28

**Authors:** Amreen Mughal, Chengwen Sun, Stephen T. O’Rourke

**Affiliations:** Department of Pharmaceutical Sciences, North Dakota State University, Fargo, ND, United States

**Keywords:** apelin, coronary artery, nitric oxide, vasorelaxation, BKCa channels

## Abstract

Apelin-APJ receptor signaling regulates vascular tone in cerebral and peripheral arteries. We recently reported that apelin inhibits BK_Ca_ channel function in cerebral arteries, resulting in impaired endothelium-dependent relaxations. In contrast, apelin causes endothelium-dependent relaxation of coronary arteries. However, the effects of apelin on BK_Ca_ channel function in coronary arterial myocytes have not yet been explored. We hypothesized that apelin-APJ receptor signaling does not have an inhibitory effect on coronary arterial BK_Ca_ channels and hence does not alter nitric oxide (NO)-dependent relaxation of coronary arteries. Patch clamp recording was used to measure whole cell K^+^ currents in freshly isolated coronary smooth muscle cells. Apelin had no effect on the increases in current density in response to membrane depolarization or to NS1619 (a BK_Ca_ channel opener). Moreover, apelin did not inhibit NO/cGMP-dependent relaxations that required activation of BK_Ca_ channels in isolated coronary arteries. Apelin-APJ receptor signaling caused a marked increase in intracellular Ca^2+^ levels in coronary arterial smooth muscle cells, but failed to activate PI3-kinase to increase phosphorylation of Akt protein. Collectively, these data provide mechanistic evidence that apelin has no inhibitory effects on BK_Ca_ channel function in coronary arteries. The lack of inhibitory effect on BK_Ca_ channels makes it unlikely that activation of APJ receptors in coronary arteries would adversely affect coronary flow by creating a vasoconstrictive environment. It can be expected that apelin or other APJ receptor agonists in development will not interfere with the vasodilator effects of endogenous BK_Ca_ channel openers.

## Introduction

Apelin is a vasoactive peptide found in many organs and tissues including, for example, adipose tissue (Boucher et al., 2005), atria (Földes et al., 2003), blood vessels (Kleinz and Davenport, 2004), lungs, kidneys, brain (Folino et al., 2015; Yan et al., 2020). Apelin acts via binding to G-protein-coupled-receptors known as APJ receptors, which are widely expressed throughout the cardiovascular system (Katugampola et al., 2001; Kleinz et al., 2005; Pitkin et al., 2010; Luo et al., 2018). An increasing body of evidence indicates that the apelin-APJ receptor signaling system regulates vascular function in mammalian arteries and veins (Gurzu et al., 2006; Jia et al., 2007; Salcedo et al., 2007; Wang et al., 2016; Nagano et al., 2019), including those from humans (Katugampola et al., 2001; Kleinz and Davenport, 2004; Maguire et al., 2009; Schinzari et al., 2017). Based on their generally favorable hemodynamic properties, apelin and apelin-based mimetics are currently in development for the treatment of several cardiovascular disorders, including heart failure, pulmonary hypertension, and ischemia/reperfusion injury (Wang et al., 2013; Brame et al., 2015; Gerbier et al., 2017; Mughal and O'Rourke, 2018).

BK_Ca_ channels play a central role in maintaining membrane potential and excitability in vascular smooth muscle cells (Tykocki et al., 2017; Dopico et al., 2018). Opening of BK_Ca_ channels leads to efflux of potassium ions, cell membrane hyperpolarization, and smooth muscle relaxation (Brayden and Nelson, 1992; Nelson and Quayle, 1995). Several endogenous vasodilator molecules, including NO and certain hyperpolarizing factors released from endothelial cells, exert their smooth muscle relaxant effects by targeting BK_Ca_ channels (Bolotina et al., 1994; Campbell et al., 1996; Mistry and Garland, 1998; Earley et al., 2005; Zhang et al., 2012). Similarly, pharmacologic agents that increase BK_Ca_ channel opening cause vascular smooth muscle relaxation and vasodilation (Ghatta et al., 2006; Jackson, 2017). By contrast, substances that interfere with BK_Ca_ channel opening create an environment that favors vasoconstriction (Tang et al., 2017; Dogan et al., 2019). Thus, modulation of BK_Ca_ channel activity is a critical mechanism for regulating blood vessel diameter and flow.

It is widely recognized that the effects of vasoactive agents may vary depending on the anatomic location of the blood vessel under investigation and/or the experimental conditions. Previous work from our laboratory demonstrates that APJ receptors are expressed in coronary and cerebral arteries (Mughal et al., 2018a; Mughal et al., 2018b). In coronary arteries, apelin acts on endothelial APJ receptors to cause nitric oxide (NO) release and endothelium-dependent relaxation (Mughal et al., 2018a). By contrast, apelin causes neither vasoconstriction nor vasorelaxation in cerebral arteries, despite the presence of APJ receptors on both endothelial cells and myocytes (Mughal et al., 2018b). However, activation of cerebral arterial smooth muscle APJ receptors by apelin inhibits BK_Ca_ channel function, thereby impairing arterial relaxation induced by NO and an endothelium-dependent hyperpolarization (EDH) pathway (Mughal et al., 2018b; Mughal et al., 2020).

Direct effects of apelin on BK_Ca_ channels in coronary arterial smooth muscle cells, and on the functional response to endogenous mediators that act via BK_Ca_ channel activation, are presently unknown. Thus, the present study was designed to increase our understanding of the apelin-APJ receptor signaling pathway in coronary arteries. Here, we tested the hypothesis that apelin-APJ receptor signaling does not have an inhibitory effect on coronary arterial BK_Ca_ channels and hence does not alter nitric oxide (NO)-dependent relaxation of coronary arteries.

## Materials and Methods

### Animals and Tissue Preparation

Twelve-week-old male Sprague-Dawley rats were used for all experiments (Envigo RMS, Indianapolis, IN, United States). Rats were housed at 22 ± 2°C (32% humidity) on a 12–12 h light-dark cycle and provided with food and water ad libitum. All animal protocols were approved by the North Dakota State University Institutional Animal Care and Use Committee. Rat hearts were isolated from animals anesthetized with isoflurane and placed into ice-cold physiological salt solution (PSS). Both left and right epicardial coronary arteries and associated branches were dissected and cleaned of surrounding tissues.

### Smooth Muscle Cell Isolation

Smooth muscle cells were isolated by enzymatic digestion as described previously (Mughal et al., 2018b). Briefly, cell isolation was performed in isolation solution of the following composition (in mM): 60 NaCl, 80 Na-glutamate, 5 KCl, 2 MgCl_2_, 10 glucose, and 10 HEPES (pH 7.2). Arterial segments were first incubated in 1.2 mg/ml papain (Worthington, Lakewood, NJ, United States) and 2.0 mg/ml dithioerythritol (Sigma Aldrich, St. Louis, MO, United States) for 17 min at 37°C, and then in 0.8 mg/ml type II collagenase (Worthington) for 12 min at 37°C. After digestion, the segments were washed with ice-cold isolation solution and triturated with a glass pipette to liberate single smooth muscle cells. Isolated cells were kept on ice and were used within 6–8 h following isolation for electrophysiology.

### Electrophysiological Recording

Freshly isolated arterial myocytes were placed into a recording chamber (Warner Instruments, Hamden, CT, United States) and whole cell current recordings were performed using our previously described protocol (Mughal et al., 2018a). Briefly, cells were perfused with a bath solution containing (in mM) 145 NaCl, 5.4 KCl, 1.8 CaCl_2_, 1 MgCl_2_, 5 HEPES, 10 glucose; pH 7.4 (NaOH). The internal pipette solution contained (in mM) 145 KCl, 5 NaCl, 0.37 CaCl_2_ (estimated free Ca^2+^ = 172 nM), 2 MgCl_2_, 10 HEPES, 1 EGTA, 7.5 glucose; pH 7.2 (KOH). Whole cell K^+^ currents were recorded at the room temperature (25°C) by stepping in 10 mV increments from a holding potential of −60 to +80 mV using an AxoPatch 200B amplifier equipped with an Axon CV 203BU headstage (Molecular Devices). The patch electrodes (3–4 MΩ) were pulled from 1.5 mm borosilicate glass capillaries. Voltage-activated currents were filtered at 2 kHz and digitized at 10 kHz, and capacitative and leakage currents were subtracted digitally. Series resistance and total cell capacitance were calculated from uncompensated capacitive transients in response to 10 ms hyperpolarizing step pulses (5 mV) or obtained by adjusting series resistance and whole-cell capacitance using the Axopatch 200B amplifier control system. All drugs were diluted in fresh bath solution and perfused into the experimental chamber. Data were collected and analyzed with pCLAMP 10.0 software (Molecular Devices, Sunnyvale, CA, United States). All currents were expressed as current density (current at the end of each voltage step divided by cell membrane capacitance).

### Vascular Function Studies

Coronary arterial segments (120–150 μm; 1.2 mm in length) were suspended in wire myograph chambers (DMT, Aarhus, Denmark) for isometric tension recording, as described previously (Mughal et al., 2018a). Briefly, the tissues were bathed in physiologic salt solution (PSS) of the following composition [(in mM): 119 NaCl, 15 NaHCO_3_, 4.6 KCl, 1.2 MgCl_2_, 1.2 NaH_2_PO_4_, 1.5 CaCl_2_ and 5.5 glucose] at 37°C. Vessel reactivity was confirmed using KCl (60 mM) and presence of endothelium was tested using the endothelium-dependent vasodilator, acetylcholine (ACh; 10^−6^ M). Vessels that showed >90% relaxation to ACh were considered endothelium-intact vessels. Responses to the vasodilators used in this study were obtained in arterial rings contracted with 5-hydroxytryptamine (5-HT; 10^−7^ M) to a sub-maximal level [∼30% of maximum contraction induced by KCl (60 mM)]. Paired experiments with inhibitors were performed in control rings isolated from the same animals.

### Intracellular Ca^2+^ Imaging

Smooth muscle cells were plated in eight-well borosilicate cover glass chambers (ThermoFisher Scientific, Waltham, MA, United States) and allowed to adhere to the glass surface (at 37°C) in endothelial growth media with 5% FBS (Promocells, Heidelberg, Germany) for 60 min. After incubation, cells were thoroughly washed with Hanks’ balanced salt solution and incubated for 60 min with fluo-4 AM (5 μM). At the end of incubation, cells were washed again and perfused with Hanks’ balanced salt solution. Real-time Ca^2+^ imaging was performed using a Zeiss confocal laser-scanning microscope (Carl Zeiss, Germany) equipped with a 40× numerical aperture oil immersion lens (with excitation at 488 nm and emission at 515 nm). All experiments were carried out at room temperature in Hanks’ balanced salt solution within 6–8 h following isolation. The effect of apelin (10^−7^ M) on intracellular Ca^2+^ levels, in the absence and presence of the APJ receptor antagonist (Lee et al., 2005), F13A (10^−7 ^M × 5 min), was determined in paired experiments. Smooth muscle cells were freshly isolated from three animals (each animal on different day) and three to four cells were analyzed for each treatment from each animal.

### Western Immunoblotting

Protein expression was performed according to our previously published method for coronary arteries with minor modification (Mughal et al., 2018a). Briefly, isolated coronary arteries were treated with apelin (10^−7^ M) for different durations (5, 10, 15 and 30 min) at 37°C. Then arterial segments were snap frozen and protein was isolated. Protein estimation was performed using a Pierce BCA protein estimation kit (ThermoFisher Scientific, Waltham, MA, United States). Aliquots of supernatant containing equal amounts of protein (40 μg) were separated on 7.5–10% polyacrylamide gel by SDS-PAGE, and electroblotted onto a PVDF membrane (Bio-Rad Laboratories, Hercules, CA, United States). Blots were blocked with 5% nonfat dry milk in Tris-buffered saline (TBS, pH 7.4) and incubated with primary antibodies, followed by secondary antibody linked to horseradish peroxidase (1:100; Cell Signaling). The blots were first probed with Akt antibody (1:500 dilution; Cell Signaling, Denver, MA, United States), followed by chemical stripping and reprobing with phospho-Akt (p-Akt) antibody (1:500 dilution; Cell Signaling, Denver, MA, United States). To ensure equal loading, the blots were probed for β-actin using an anti-actin antibody (1:500; Santa Cruz Biotechnology Inc.). Immuno-detection was performed using an enhanced chemiluminescence light detection kit (ThermoFisher Scientific).

### Drugs

The following drugs were used: acetylcholine (ACh), diethylamine NONOate (DEA NONOate), 5-HT, and NS1619 (Sigma Chemical, St. Louis, MO, United States); iberiotoxin (IBTx) and eight-Bromo-cGMP (8-Br-cGMP) (Tocris, Ellisville, MO); apelin-13 and F13A (H-Gln-Arg-Pro-Arg-Leu-Ser-His-Lys-Gly-Pro-Met-Pro-Ala-OH trifluoroacetate salt) (Bachem, Torrance, CA, United States). Drug solutions were prepared fresh daily, kept on ice, and protected from light until used. All drugs were dissolved initially in double-distilled water. Drugs were added to the myograph chambers in volumes not greater than 0.02 ml. Drug concentrations are reported as final molar concentrations in the myograph chamber.

### Data Analysis

Relaxation responses are expressed as a percent of the initial tension induced by 5-HT (10^−7^ M). EC_50_ values (drug concentration that produced 50% of its own maximal response) were determined, converted to their negative logarithm, and expressed as -log molar EC_50_ (pD_2_). For Ca^2+^ imaging studies, the amplitude of Fluo-4 AM fluorescence was calculated by peak fluorescence intensity (F) divided by baseline intensity (F0). Results are expressed as means ± SEM, and n refers to the number of animals from which blood vessels and/or smooth muscle cells were taken, unless otherwise stated. All data sets were tested for normal distribution using the Shapiro–Wilk test. Values were compared by Student’s t-test or one-way ANOVA using Tukey’s test as post-hoc analysis for paired or unpaired parametric distributed observations, as appropriate, to determine significance between groups. Mann-Whitney test was applied for non-parametric distributed observations. Values were considered significantly different when *p* < 0.05.

## Results

### Electrophysiology Studies (Whole Cell K^+^ Currents)

**FIGURE 1:**
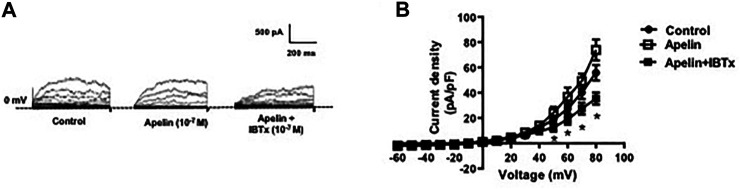
Effect of apelin on whole cell K^+^ currents in coronary arterial myocytes. Whole cell K^+^ currents were recorded in freshly isolated myocytes in response to successive voltage pulses of 800 ms duration, increasing in 10 mV increments from −60 to +80 mV with or without apelin (10^−7^ M) or apelin plus IBTx (10^-7^ M). **(A)** Representative traces showing the current recordings from smooth muscle cell in the absence and presence of apelin alone or apelin plus IBTx. **(B)** Summary I–V curves of whole cell K^+^ currents at baseline (control) and after treatment with apelin (10^−7^ M) alone or in the presence of IBTx (10^−7^ M). Dotted line represents zero current level (at 0 mV). Values are presented as the mean ± S. E. M. (*n* = 6). *p < 0.05 vs. control current density.

**FIGURE 2:**
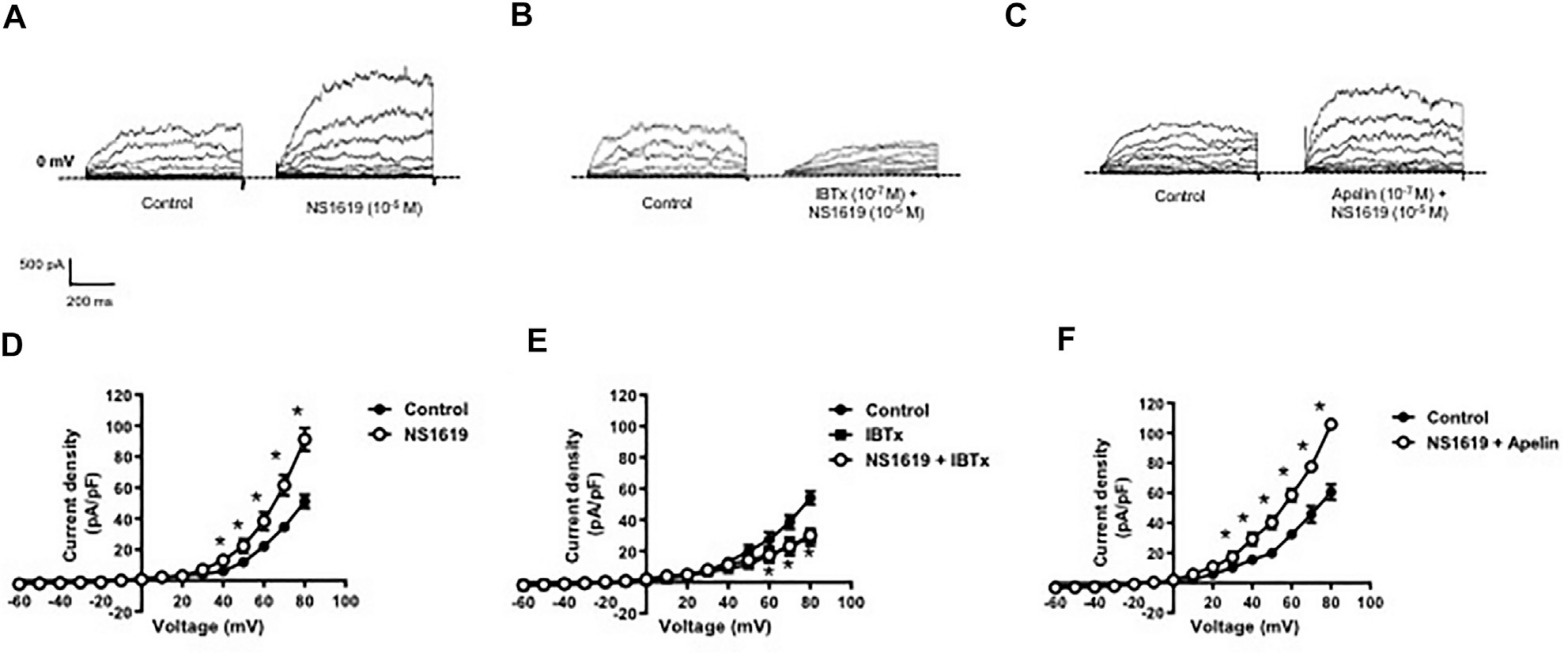
Effect of apelin on NS1619-induced BK_Ca_ currents in coronary arterial myocytes. Whole cell BK_Ca_ currents induced in response to NS1619 (10^-5^ M) were recorded in freshly isolated coronary arterial myocytes. Representative traces showing the current recordings from smooth muscle cells after treatment with **(A)** NS1619 (10^−5^ M, 2 min) alone or **(B)** in the presence of IBTx (10^−7^ M, 5 min) or **(C)** apelin (10^−7^ M, 5 min). **(D**–**F)** Summary I–V curves of BK_Ca_ currents at baseline and after treatment with **(D)** NS1619 (10^−5^ M) alone or in the presence of **(E)** IBTx (10^−7^ M) or **(F)** apelin (10^−7^ M). Dotted line represents zero current level (at 0 mV). Values are presented as the mean ± S.E.M. (*n* = 6–7). *p < 0.05 vs. control current density.

Whole cell K^+^ currents were recorded in freshly isolated smooth muscle cells from coronary arteries. Superfusion of apelin (10^−7^ M, 5 min) had no inhibitory effect on whole cell K^+^ currents, but addition of the selective BK_Ca_ channel blocker, iberiotoxin (10^−7^ M, 5 min), significantly reduced the current density (Figure 1). Incubation of coronary myocytes with NS1619 (10^−5^ M, 3 min), a selective BK_Ca_ channel opener, caused a significant increase in the current density (Figures 2A,D). The NS1619-induced increase in current density was markedly attenuated by iberiotoxin (Figures 2B,E), whereas apelin had no inhibitory effect (Figures 2C,F).

### Vascular Relaxation Studies (NO/cGMP-Induced Relaxation)

**FIGURE 3:**
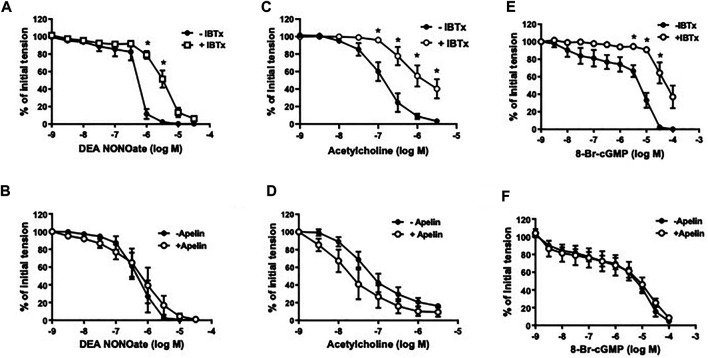
Effect of apelin and iberiotoxin on NO-cGMP dependent vasorelaxation of coronary arteries. Log concentration-response curves in producing relaxation of coronary arteries for DEA NONOate **(A,B)**, acetylcholine **(C,D)** or 8-Bromo-cGMP **(E,F)** in the absence and presence of IBTx (10^−7^ M) **(A, C, E)** or apelin (10^−7^ M) **(B, D, F)**. Values are presented as the mean ± S.E.M. (*n* = 4–6). *p < 0.05 vs. control (in the absence of inhibitor).

In coronary arterial segments contracted with five-HT (10^−7^ M), the NO donor, DEA NONOate (DEA, 10^−9^ − 3 × 10^−5^ M), caused concentration-dependent relaxation (Figure 3). Iberiotoxin (10^−7^ M) caused a significant rightward shift in the DEA concentration-response curve (Figure 3A); however, DEA-induced relaxations were unaffected in the presence of apelin (10^−7^ M) [pD_2_ = 6.38 ± 0.20 vs. 6.15 ± 0.28 in the absence and presence of apelin, respectively; *p* > 0.05 (*n* = 5)] (Figure 3B). Similarly, relaxations induced by acetylcholine (10^−9^ − 3 × 10^−6^ M), an endothelium-dependent vasodilator that acts via NO release from endothelial cells, were significantly inhibited by iberiotoxin (Figure 3C), but not by apelin [pD_2_ = 7.16 ± 0.17 vs. 7.38 ± 0.30 in the absence and presence of apelin, respectively; *p* > 0.05 (*n* = 6)] (Figure 3D). The stable cGMP analog, 8-Bromo-cGMP [8-Br-cGMP (10^−9^ – 10^−4^ M)], also caused concentration-dependent relaxation of isolated coronary arteries. The response to 8-Br-cGMP was significantly attenuated in the presence of iberiotoxin (Figure 3E), but not apelin [pD_2_ = 5.25 ± 0.16 vs. 5.41 ± 0.44 in the absence and presence of apelin, respectively; *p* > 0.05 (n = 4)] (Figure 3F).

### Intracellular Signaling Studies (Ca_i_^2+^ and PI3 Kinase Activity)

**FIGURE 4:**
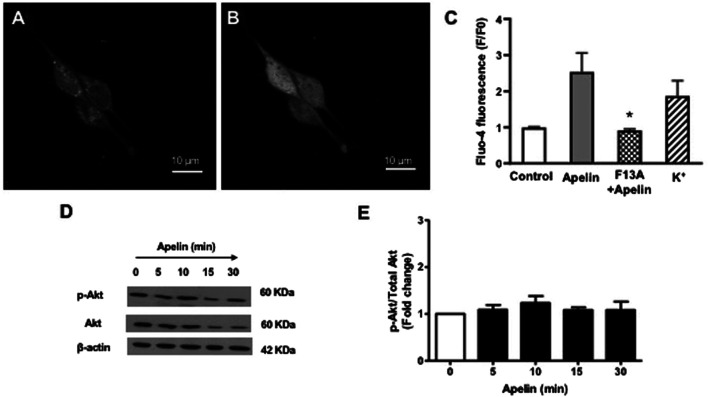
Effect of apelin-APJ signaling on coronary intracellular Ca^2+^ levels and PI3-kinase activity. Representative images of freshly isolated coronary arterial smooth muscle cells loaded with Fluo-four AM (5 µM) **(A)** before and **(B)** after exposure to apelin (10^−7^ M). **(C)** Bar graph summarizing changes in fluorescence (F/F0) in response in apelin alone (10^−7^ M) or in the presence of F13A (10^−7^ M); 60 mM K^+^ was used as a positive control for the experiments (*n* = 3). **(D)** Representative blots showing changes in phosphorylation of Akt (p-Akt) or Akt (total) protein expression in a time-dependent manner following treatment with apelin (10^−7^ M). **(E)** Summary data showing effect of apelin on PI3-kinase activity expressed as a ratio of p-Akt/Akt protein levels (*n* = 3). Values are presented as the mean ± S.E.M. *p < 0.05 vs. apelin alone; **p < 0.05 vs. control.

The functional status of coronary smooth muscle APJ receptors was assessed using intracellular calcium imaging. Apelin (10^-7^ M) caused a robust increase in intracellular Ca^2+^ levels in freshly isolated smooth muscle cells (Figures 4A–C). The apelin-induced rise in intracellular Ca^2+^ was comparable to that observed by depolarization with 60 mM K^+^, which served as a positive control (Figure 4C). The APJ receptor antagonist, F13A (10^-7^ M, 5 min), abolished the apelin-induced response, thus confirming functional activation of smooth muscle APJ receptors in response to apelin in coronary arteries (Figure 4C).

Since apelin is known to increase PI3 kinase activity in cerebral arteries (Modgil et al., 2013), experiments were performed to evaluate the effect of apelin on PI3 kinase activity in coronary arteries. PI3-kinase activity was detected by measuring the ratio of phosphorylated Akt to total Akt, using immunoblot analysis. Apelin (10^-7^ M) treatment caused no significant change in PI3-kinase activity in coronary arteries, as indicated by unchanged P-Akt/total Akt ratio (Figures 4D,E).

## Discussion

The present study was designed to evaluate the effects of apelin on BK_Ca_ channels in coronary arterial smooth muscle cells. The key findings are: 1) apelin has no inhibitory effect on BK_Ca_ currents in coronary arterial smooth muscle cells; and 2) apelin does not inhibit NO/cGMP-induced relaxations, which are dependent on BK_Ca_ channels in coronary arteries (Darkow et al., 1997; Tunstall et al., 2011). These results are in striking contrast to our previous findings in cerebral arteries (Mughal et al., 2018b; Mughal et al., 2020), and clearly demonstrate that distinct molecular mechanisms underly the vasomotor effects of apelin in coronary and cerebral arteries.

BK_Ca_ channels are expressed in vascular smooth muscle cells from rat cerebral and coronary arteries (Modgil et al., 2013; Mughal et al., 2018a; Mughal et al., 2018b). In cerebral arteries, apelin inhibits smooth muscle BK_Ca_ channels and thereby impairs endothelium-dependent relaxations mediated by NO and an EDH pathway (Mughal et al., 2018b; Mughal et al., 2020). The results of the present study demonstrate that in coronary arterial myocytes apelin failed to inhibit increases in BK_Ca_ current density induced by membrane depolarization or by pharmacological activation with NS1619. Consistent with these electrophysiological findings, apelin was without effect on coronary arterial relaxation caused by endothelium-derived NO (i.e. acetylcholine), exogenously administered NO (i.e., DEA NONOate), and the stable, cell permeable cGMP analogue, 8-Br-cGMP. The absence of an inhibitory effect of apelin cannot be attributed to the lack of involvement of BK_Ca_ channels in NO/cGMP pathway-induced relaxation of rat coronary arteries since iberiotoxin, a potent and selective BK_Ca_ channel blocker (Kaczorowski and Garcia., 2016; Su et al., 2016), markedly impaired the responses to ACh, DEA, and 8-Br-cGMP. That apelin had no effect on these responses also cannot be explained by an absence of APJ receptors, which are indeed expressed in rat coronary arterial smooth muscle cells (Mughal et al., 2018a). Taken together, these data indicate that APJ receptors are not effectively coupled to BK_Ca_ channels in coronary arterial smooth muscle cells.

One possible explanation for the differential effect of apelin in cerebral vs coronary arterial smooth muscle cells could be related to the intracellular signaling pathways stimulated in response to apelin-APJ receptor activation. Binding of apelin to APJ receptors in cerebral arterial smooth muscle cells stimulates the PI3 kinase/Akt signaling pathway, which is required for apelin-induced inhibition of BK_Ca_ channel function (Modgil et al., 2013). That apelin did not activate the PI3K/Akt-signaling pathway in coronary arterial smooth muscle cells provides a plausible mechanistic explanation as to why APJ receptors may not be efficiently linked to BK_Ca_ channels in coronary myocytes; however, the possibility that another intracellular signaling pathway plays a role in the lack of inhibitory effect of apelin on BK_Ca_ channel function cannot be ruled out. The absence of effect of apelin on BK_Ca_ channels and PI3 kinase/Akt signaling is not due to an inability to activate APJ receptor signaling in coronary arterial smooth muscle cells, since apelin caused a rapid and robust increase in intracellular Ca^2+^ levels. The apelin-induced rise in intracellular Ca^2+^ levels was attenuated by the APJ receptor antagonist, F13A, thus confirming that smooth muscle APJ receptors are indeed responsive to apelin in coronary arteries. It is also worth noting that although apelin caused an increase in intracellular Ca^2+^ levels in coronary smooth muscle cells, it does not cause contraction of isolated coronary arteries (Mughal et al., 2018a). This opens the possibility that apelin/APJ receptor signaling in coronary arterial smooth muscle cells may be involved in other cellular functions beyond the regulation of vascular tone.

The coronary circulation is controlled by multiple regulatory systems, including, for example, metabolic autoregulation, the sympathetic nervous system, circulating vasoactive molecules, and mediators produced by endothelial cells located within the blood vessel wall (Goodwill et al., 2017). The extent to which such systems play a role in regulating vasomotor tone varies between epicardial arteries (conductance vessels) and small arteries and arterioles (resistance vessels and microcirculation). For example, NO is a principal regulator of epicardial coronary vasomotor tone, whereas metabolic autoregulation and EDH pathways play a more prominent role in smaller resistance vessels and the microcirculation (Deussen et al., 2012; Ellinsworth et al., 2016; Goodwill et al., 2017). The present findings show a lack of inhibitory effect of apelin/APJ receptor signaling on the NO/cGMP/BK_Ca_ channel pathway in epicardial arteries, but whether or not this is true for other regions of the coronary circulation remains to be determined.

As with all biological studies, there are some limitations that should be considered. Although pre-constricted coronary arterial rings were used to investigate the effects of apelin on the vasodilators used in this study, it is possible that the vascular effects of apelin may differ in unstimulated arterial rings with intrinsic myogenic tone. In addition, our present findings are limited to arteries obtained from healthy adult male rats. It is well known that responses to vasoactive mediators may vary depending on the age and sex of the animals, as well as in pathological conditions. Thus, future studies with female rats, various age groups, and appropriate models of cardiovascular disease will be needed to further enhance our understanding of the vasomotor effects of apelin.

The results of the present study build on our knowledge of the actions of apelin in coronary arteries. The ability of apelin to cause endothelium-dependent relaxation (Mughal et al., 2018a), coupled with a lack of inhibitory effect on BK_Ca_ channels makes it unlikely that activation of APJ receptors in coronary arteries would adversely affect epicardial coronary flow by creating a vasoconstrictive environment. It could be expected that apelin or other APJ receptor agonists in development will not interfere with the vasodilator effects of endogenous BK_Ca_ channel openers, nor would these agents be likely to elicit coronary vasospasm, which has been hypothesized to be related to reduced K conductance in coronary artery smooth muscle cells (Dick and Tune, 2010).

## Data Availability

The raw data supporting the conclusion of this article will be made available by the authors, without undue reservation.
